# Neutralizing Antibodies to Severe Fever With Thrombocytopenia Syndrome Virus Among Survivors, Non-Survivors and Healthy Residents in South Korea

**DOI:** 10.3389/fcimb.2021.649570

**Published:** 2021-03-23

**Authors:** Jeong Rae Yoo, Jae-Yong Kim, Sang Taek Heo, Jihye Kim, Hyeong-Jun Park, Joo-Yeon Lee, Hee-Young Lim, Woo-Jung Park, Nam-Hyuk Cho, Jung Mogg Kim, Jae-Hwan Nam, Keun Hwa Lee

**Affiliations:** ^1^ Department of Infectious Diseases, Jeju National University College of Medicine, Jeju, South Korea; ^2^ Department of Medical and Biological Sciences, The Catholic University of Korea, Bucheon, South Korea; ^3^ Department of Medical Nutrition, Graduate School of East-West Medical Science, Kyung Hee University, Yongin, South Korea; ^4^ Division of Emerging Infectious Diseases and Vector Research, National Institute of Health, Korea Center for Disease Control and Prevention, Osong, South Korea; ^5^ Department of Microbiology, Seoul National University College of Medicine, Seoul, South Korea; ^6^ Department of Microbiology, Hanyang University College of Medicine, Seoul, South Korea

**Keywords:** SFTSV, neutralizing antibodies, survivors, non-survivors, healthy residents, South Korea

## Abstract

Severe fever with thrombocytopenia syndrome (SFTS), a newly emerging tick-borne viral disease, has been detected in Asia since 2009, and person-to-person transmission is possible. SFTS is characterized by atypical signs, including mild to severe febrile illness similar to that associated with hemorrhagic fever, with 16.2 to 30% mortality. We found that the titers of neutralizing antibodies, play an important role in protective immunity, to SFTS virus (SFTSV) in survivors and healthy residents who lived in endemic areas and who were positive for SFTSV IgG, were higher than those in non-survivor patients. Moreover, the titers were maintained in surviving patients and healthy residents but not in non-surviving patients in South Korea.

## Introduction

Severe fever with thrombocytopenia syndrome (SFTS), a new tick-borne viral disease with a high mortality rate, was first reported in China in 2009, South Korea in 2010 Japan in 2013, Vietnam in 2017, Myanmar in 2018, and Taiwan in 2019 (Yu et al., 2011; Takahashi et al., 2014; Kim et al., 2018; Tran et al., 2019; Peng et al., 2020; Win et al., 2020). Most SFTS virus (SFTSV) infections occur *via* bites from the tick *Haemaphysalis longicornis*; however, SFTSV transmission can also occur through close contact with an infected patient (Yoo et al., 2017). SFTS is characterized by acute high fever, thrombocytopenia, leukopenia, elevated serum hepatic enzyme levels, gastrointestinal symptoms, and multiorgan failure and has a 16.2 to 30% mortality rate (Yu et al., 2011; Takahashi et al., 2014; Peng et al., 2020). Atypical signs and symptoms as well as asymptomatic SFTS infections have also been identified in patients, and SFTS patients in China have been shown to produce and maintain long-lasting neutralizing antibodies to SFTSV (Huang et al., 2016; Yoo et al., 2017; Kim et al., 2018).

In this study, we briefly investigated the levels of neutralizing antibodies to SFTSV in serum among surviving patients, non-surviving patients, and healthy residents living in endemic areas and who were positive for SFTSV IgG antibody in South Korea from 2013 to 2019 (Yoo et al., 2019). We found that the titers of neutralizing antibodies to SFTSV maintained in surviving patients and healthy residents were higher than those detected in non-surviving patients.

## Materials and Methods

To investigate the levels of neutralizing antibodies to SFTSV, we collected 19 serum samples from 11 patients (non-surviving patients; *n*=6, mean age: 73.3 and surviving patients; *n*=5, mean age: 68.8), which were laboratory confirmed at Jeju National University from May 2013 to October 2019, Jeju, South Korea, and 7 serum samples from 5 healthy residents (mean age: 69.8) living in endemic areas who were positive for SFTSV IgG from November 2015 to April 2017 (Yoo et al., 2019) (**Table 1**). This study was approved by the Institutional Review Board (IRB) of Jeju National University Hospital.

**Table 1:** Baseline characteristics of the SFTS patients and healthy residents.

Patient	Age (year)/Sex	Date of sampling (onset)	CCI*	Route of infection	Outcome	ANC (/μl)	PLT (/10^3^ μl)	AST (IU/L)	ALT (IU/L)	CK (IU/L)	LDH (IU/L)	aPTT (/sec)	MODS^†^	FRNT50 titer
Jeju-P01	73/M	May-16-13 (May-02-13)	0	Tick	Death	724	30	392	136	4,377	2,177	74	19	6.57
Jeju-P04	62/M	Jun-13-13 (Jun-05-13)	0	Tick	Death	815	55	524	152	588	1,481	51	16	6.22
Jeju-P16	74/M	Jun-10-15 (Jun-07-15)	1	Tick	Death	1530	47	157	56	345	1,071	53	13	0
Jeju-P48	71/M	May-02-18 (April-07-18)	2	Tick	Death	4,280	214	51	16	359	N/A	26	11	7.73
Jeju-P49-1	81/F	Jun-12-18 (Jun-06-18)	1	Tick	Recovery	250	64	35	33	1,030	615	35	3	3.40
-2		Jun-14-18				190	22	368	112	862	2242	48	4	2.17
-3		Jun-16-18				120	38	314	118	339	1353	31	4	0.60
-4		Jun-18-18				1,000	74	265	155	111	1,177	30	3	9.89
-5		Jun-20-18				2,010	147	258	185	69	854	N/A	2	2.80
Jeju-P55	68/M	Oct-11-18 (Oct-08-18)	1	Tick	Death	317	55	713	130	455	8,559	713	12	0
Jeju-P57-1	37/F	Oct-21-18 (Oct-21-18)	0	Patient	Recovery	1,100	168	33	16	62	380	33	1	2.48
-2		Dec-24-18				N/A	N/A	N/A	N/A	N/A	N/A	N/A	N/A	105.3
-3		Oct-17-19				N/A	N/A	N/A	N/A	N/A	N/A	N/A	N/A	123.4
Jeju-P63	92/F	July-05-19 (Jun-06-19)	1	Tick	Death	1,230	90	34	84	323	1,013	34	7	1.11
Jeju-P64	64/M	Aug-12-19 (Aug-07-19)	0	Tick	Recovery	260	62	36	78	N/A	571	36	2	0
Jeju-P66-1	60/M	Aug-28-19 (Aug-24-19)	1	Tick	Recovery	890	128	33	45	106	657	33	1	10.25
-2		Aug-30-19				450	108	80	56	589	172	30	1	5.86
Jeju-P70-1	70/F	Oct-11-19 (Oct-10-19)-	0	Tick	Recovery	3,460	79	38	21	N/A	682	38	10	13.53
-2		Oct-22-19				12,370	76	21	21	N/A	647	34	11	34.82
Jeju-H10	77/F	Nov-24-15 (N/A)	3	N/A	Healthy	N/A	N/A	N/A	N/A	N/A	N/A	N/A	N/A	31.52
Jeju-H13	67/M	Nov-24-15 (N/A)	0	N/A	Healthy	N/A	N/A	N/A	N/A	N/A	N/A	N/A	N/A	24.88
Jeju-H17	78/M	Nov-24-15 (N/A)	0	N/A	Healthy	N/A	N/A	N/A	N/A	N/A	N/A	N/A	N/A	24.25
Jeju-H21-1	56/F	Nov-11-15 (N/A)	0	N/A	Healthy	N/A	N/A	N/A	N/A	N/A	N/A	N/A	N/A	129.4
-2		July-28-16				N/A	N/A	N/A	N/A	N/A	N/A	N/A	N/A	67.67
-3		April-30-17				N/A	N/A	N/A	N/A	N/A	N/A	N/A	N/A	96.84
Jeju-H22	71/F	Nov-24-15 (N/A)	0	N/A	Healthy	N/A	N/A	N/A	N/A	N/A	N/A	N/A	N/A	104.7

M, male; F, female; *CCI, Charlson comorbidity index score, predicts the 10-year mortality for a patient who may have a range of comorbid conditions; SFTS, severe fever with thrombocytopenia syndrome; ANC, absolute neutrophil count; PLT, platelet; AST, aspartate aminotransferase; ALT, alanine aminotransferase; CK, creatinine kinase; LDH, lactate dehydrogenase; aPTT, activated partial thromboplastin time; ^†^MODS, multiple organ dysfunction syndrome score, constructed using simple physiologic measures of dysfunction in six organ systems, mirrors organ, and correlates strongly with the ultimate risk of intensive care unit mortality and hospital mortality; N/A, not applicable.

For the molecular diagnosis of SFTSV, RNA was extracted from the stored patient serum using a QIAamp Viral RNA Mini Kit (QIAGEN, Hilden, Germany). Real-time RT-PCR was performed to amplify the partial small (S) segment of the viral RNA from the stored serum and confirm SFTSV infection (Zhang et al., 2012; Yun et al., 2015). Real-time RT-PCR showed that all the patients (*n*=11) were positive for SFTSV, and 6 healthy residents were negative for SFTSV. Antibody detection was performed, and IgG was detected in the serum of all patients and 6 healthy residents (Yoo et al., 2019).

To determine the titers of neutralizing antibody to SFTSV in human sera, the 50% focus reduction neutralization test (FRNT50) assay was performed. Serum samples were heat inactivated at 56°C for 30 min and diluted in 2-fold increments from 1:10 to 1:320. Each dilution was mixed with an equal volume of solution containing SFTSV (1600 ffu/mL). The mixture was inoculated into Vero E6 cells prepared in 24-well plates and then incubated for 1 hour at 37°C. Culture medium was used as a control. After incubation, the cells were overlaid with 1 mL of Dulbecco’s modified Eagle’s medium (DMEM) containing 1.5% carboxymethyl cellulose, and the cells were incubated for an additional 2 days. After 2 days of incubation, cells were fixed with 4% paraformaldehyde and incubated with 500 µl/well of anti-SFTSV NP monoclonal antibodies diluted with 0.5% Triton X-100 in PBS for 90 min at room temperature (RT), followed by incubation with HRP-conjugated secondary antibodies. The visualization of foci of SFTSV-infected cells was performed using a DAB substrate kit. The plaque reduction percentage was calculated by the following formula: [(number of foci of SFTSV diluted without serum)-(number of foci of SFTSV diluted with serum)] x 100/number of foci of SFTSV diluted without serum. From this plaque reduction percentage, the FRTN50 titers were calculated by the [log(inhibitor) vs. normalized response] equation using GraphPad Prism 8.0.

For statistical analysis, data were expressed as medians and 95% confidence intervals. Neutralizing antibodies among three groups (non-surviving patients, surviving patients and healthy residents) according to the dilution factor was examined using Kruskal-Wallis test. All data were analyzed using SAS software, version 9.4 (SAS Institute, Cary, NC, USA). A *p* value < 0.05 was considered statistically significant for two-sided tests.

## Results

**Figure 1:**
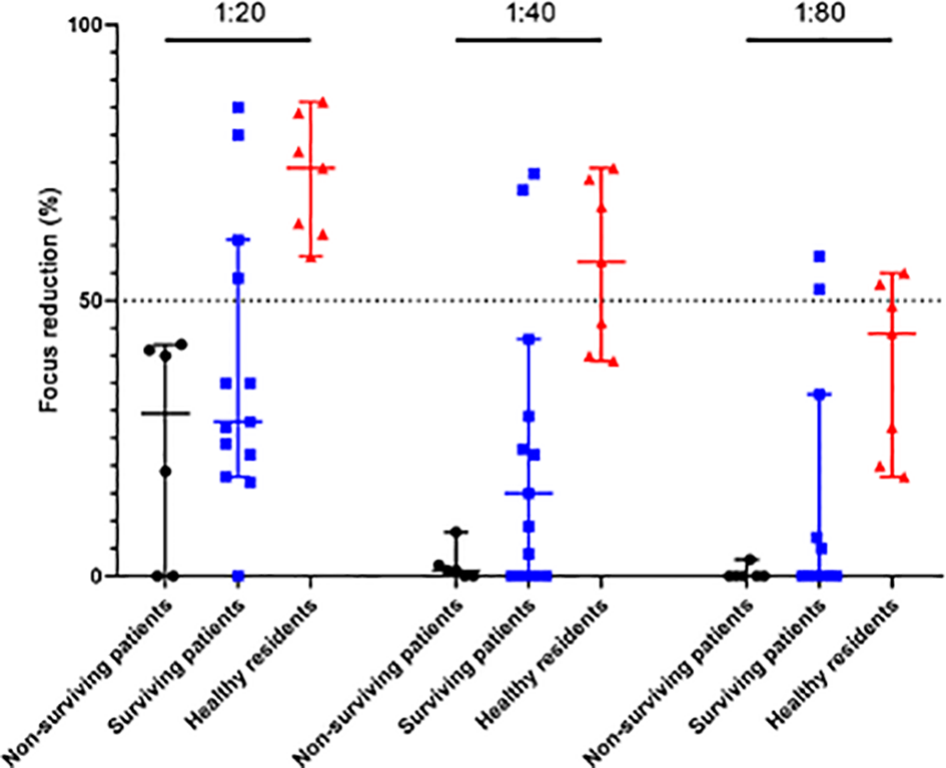
Neutralizing antibody titrations. Neutralizing antibodies to SFTSV were evaluated (FRNT50) in serum samples collected from 6 non-surviving patients, 13 serum samples from 5 surviving patients, and 7 serum samples from 5 healthy residents. Individual samples were serially diluted from 1∶20 to 1∶80. Statistically significant differences were found among surviving patients, non-surviving patients, and healthy residents (*p* <0.05 for all dilution factor).

The FRNT50 results showed that 4 surviving patients developed neutralizing antibodies against SFTSV, with titers ranging from 1:20 to 1:80, and 5 healthy residents were positive for SFTSV IgG. The healthy residents had no typical symptoms or other infectious diseases, had no history of tick bites or contact with SFTS patients during the period of sample collection, and developed neutralizing antibodies against SFTSV at titers ranging from 1:20 to 1:80. However, 6 non-surviving patients did not have well-developed neutralizing antibodies. Neutralizing antibodies to SFTSV differed significantly among surviving patients, non-surviving patients, and healthy residents regardless of dilution factor (*p* <0.05 for all dilution factor) (**Figure 1**).

Interestingly, neutralizing antibodies one surviving patient (Jeju-P57) appeared at two months after discharge and persisted for one year, and neutralizing antibodies in one healthy resident (Jeju-H21) persisted for the entire study period of 3 years (from 2015 to 2017) (**Table 1**).

These results show that long-lasting neutralizing antibodies to SFTSV is maintaining in surviving patients (Huang et al., 2016).

## Conclusion

Neutralizing antibodies can directly block viral attachment to target cells by interfering with virus-receptor interactions and induce antibody-dependent cellular cytotoxicity (ADCC), complement-dependent cytotoxicity and antibody-dependent cellular phagocytosis (ADCP) (Walker et al., 2018). A previous study showed that SFTS patients produce neutralizing antibodies to SFTSV and can last for four years with a decrease in titers and suggested that neutralizing monoclonal antibody activity blocks the interactions between glycoprotein Gn and cellular receptors (Huang et al., 2016).

In conclusion, we found that surviving patients and healthy residents living in an endemic area had high titers of long-lasting neutralizing antibodies to SFTSV compared with non-surviving patients. A previous study suggested that neutralizing monoclonal antibody activity blocks the interactions between glycoprotein Gn and cellular receptors (Huang et al., 2016). Therefore, we suggest that neutralizing antibodies may play an important role in protective immunity in surviving patients and healthy residents against SFTSV infection.

However, the limitation of this study is that we followed surviving patients for up to one year, whereas we followed healthy residents for up to 3 years and not yet study about the role of long-lasting neutralizing antibodies. Therefore, we do not know how long the neutralizing antibodies in patients last and the characteristics of the neutralizing antibodies to SFTSV.

Therefore, further clinical research on lasting neutralizing antibodies and the characteristics of these antibodies are needed, and this subject deserves further discussion, which will aid in the understanding of the pathogenesis of SFTSV infection.

## Data Availability Statement

The original contributions presented in the study are included in the article/supplementary material. Further inquiries can be directed to the corresponding authors.

## Ethics Statement

The studies involving human participants were reviewed and approved by The Institutional Review Board (IRB) of Jeju National University Hospital. The patients/participants provided their written informed consent to participate in this study.

## Author Contributions

Conceptualization: KL, J-HN, SH. Methodology: KL, JHK, J-HN, JY, SH, J-YK, N-HC. Software: J-YK, JHK. Supervision: KL, J-HN, JMK. Validation: JHK. Formal analysis: KL, JHK, J-HN, JY, SH, J-YK, H-JP, J-YL, J-YL, W-JP, N-HC. Funding acquisition: J-HN, KL. Data curation: KL, J-HN, N-HC. Writing-original draft: JY, JHK, J-YK, SH, J-HN, KL. Writing—review and editing: KL, J-HN, JHK, JMK. All authors contributed to the article and approved the submitted version.

## Funding

This work was supported by J-HN was supported by SML Biotree Group fund (2019-2021), a fund (HD20A0323) by Research of Korea Center for Disease Control and Prevention, and the research fund of Hanyang University (HY-2020).

## Conflict of Interest

The authors declare that the research was conducted in the absence of any commercial or financial relationships that could be construed as a potential conflict of interest.
